# Attitudes of female market vendors of reproductive age towards use of mobile phones and access to family planning self-care interventions in Northern Uganda: a cross-sectional study

**DOI:** 10.1186/s12911-024-02565-5

**Published:** 2024-06-12

**Authors:** Yagos Onen Walter, Pamela Atim, Derrick Amone, Alarakol Simon Peter, Geoffrey Olok Tabo

**Affiliations:** 1 Department of Library and Information Service, Medical Library, Gulu University, Kampala, Uganda; 2 Department of Public Health, Faculty of Medicine, Gulu University, Kampala, Uganda; 3 Department of Surgery, Faculty of Medicine, Gulu University, Gulu, Kampala Uganda; 4 Department of Biochemistry, Faculty of Medicine, Gulu University, Kampala, Uganda; 5 Department of Computer Science, Faculty of Science, Gulu University, Kampala, Uganda

**Keywords:** Use of mobile phones, Female of reproductive age, Family planning self-care interventions, Northern Uganda

## Abstract

**Background:**

Mobile phones are potential digital technologies for accessing family planning self-care interventions. However, their utilization could be possible if women of reproductive age have positive attitudes towards the use of this technology for healthcare purposes. This study aimed to examine the relationship between attitudes towards the use of mobile phones and access to family planning self-care interventions among female market vendors of reproductive age in northern Uganda.

**Methods:**

A cross-sectional survey design was used. Two hundred and five randomly selected female vendors from the Gulu city main market participated. A structured researcher-administered questionnaire was used to collect the data. Descriptive statistics and standard multiple regression were performed, and the data were analysed using SPSS software version 15.

**Results:**

Of the 205 participants, 112 (54.6%) reported using smartphones, and 147 (71.7%) were aware of family planning self-care interventions. Participants had moderate attitudes towards access to family planning self-care interventions (mean = 3.18), positive attitudes towards ease of use (mean = 3.31) and usefulness of mobile phones (mean = 3.30), strong positive attitudes towards privacy (mean = 4.04), and skills associated with using mobile phones (mean = 4.04). Furthermore, significant positive relationships existed between ease of use (*p* value = 0.000), skills (*p* value = 0.001), privacy (*p* value = 0.002) and access to family planning self-care interventions. There was, however, an insignificant positive relationship between mobile phone usefulness and access to family planning self-care interventions (*p* value = 0.189).

**Conclusions:**

Participants’ positive attitudes towards the use of mobile phones could lead to access to FP self-care interventions, although uncertainty about the usefulness of the use of mobile phones for accessing FP self-care interventions exists. It is therefore important for healthcare practitioners, health development partners and the government to encourage and integrate the use of mHealth into regular FP self-care services and promotional activities while targeting underserved communities in Uganda.

**Supplementary Information:**

The online version contains supplementary material available at 10.1186/s12911-024-02565-5.

## Introduction

Mobile phones are potential digital technologies for accessing family planning (FP) self-care interventions [1–3]. Increasing the use of this technology makes access to sexual and reproductive health services possible, particularly self-care interventions [4, 5]. The burden of sexual and reproductive health-related problems on the health system and women in low and middle-income countries (LMICs) is considerable. Annually, approximately 74 million women in this region have unplanned pregnancies, leading to 25 million unsafe abortions and 47,000 maternal deaths [2]. Among the interventions introduced to address some of these problems is self-care intervention. The World Health Organization defines self-care as “the ability of individuals, families and communities to promote health, prevent disease, maintain health, and cope with illness and disability with or without the support of a health care provider” [6].

Self-care interventions include a wide range of health interventions, such as drugs, diagnostics, devices, digital technologies and other related interventions; these interventions can be provided fully or partly outside formal health services and can be used with or without the direct involvement of health workers [3, 6]. In the FP domain, self-care interventions include tools that support FP needs, can be provided fully or partly outside formal health services, and can be used with or without the direct involvement of health workers [3]. These interventions include self-administration of injectables, use of ovulation predictor kits, and self-testing for pregnancy, among others [6]. Using self-care interventions allows people to promote their own health; enhance privacy, confidentiality, and anonymity; and reduce costs [2, 7]. Although there are benefits, the majority of women in LMICs are not fully exposed to FP self-care interventions [8]. In sub-Saharan Africa, few as 56% of women have their needs for FP satisfied [9]. In Uganda, the percentage of women with an unmet need for FPs is worrying. Approximately 28% of married women, 32% of unmarried women and 41% of people living with HIV have unmet needs for FP [10]. In addition, approximately 20.4% of women aged 15–49 years are in unmet need [11]. The lack of access to modern FP services is mainly attributed to a weak health system, inadequate promotion and the high cost of accessing health services [2, 3, 11]. However, the use of mobile health (mHealth) devices such as mobile phones could offer new opportunities to provide access to healthcare to people who need them most [2, 6, 12]. Understanding how attitudes towards this technology influence access to health interventions is important, as it could lead to solutions for the health challenges facing the underserved community.

In Uganda, 64.6% of women owned mobile phones. The highest ownership is among women aged 18–30 years (77.4%), followed by women aged 30 years and above (73.1%), and the lowest is among women aged 10–17 years (33.4%) [11]. Clearly, the majority of women who owned mobile phones fall within the reproductive age bracket. Thus, taking full advantage of this technology for health could improve the coverage, access and uptake of FP self-care interventions, thereby addressing the challenges of unmet needs for FP [4]. By using mobile phones, women can benefit from client-to-provider telemedicine, gain self-confidence and confidentiality [13], and be empowered to access modern FP interventions with less difficulty [8–10]. A significant number of studies on the use of mobile phones in Uganda have focused on female market vendors. However, in many of these studies, discussions surrounding the use of mobile phones have been linked to how technology economically supports women in the informal business sector [14–17]. A previous study recommended innovative and targeted FP interventions to improve the use of FP services among underserved female market vendors [18]. This group of informal workers has limited access to hospital facility-based services due to the demanding nature of their occupation [19, 20]. In Uganda, there are approximately 20,000 registered market vendors, 60% of whom are women [21]. The current study addresses this gap by examining how the attitudes of female market vendors towards the use of mobile phones influence access to FP self-care interventions. This could help inform interventions that can leverage the use of mobile phones to facilitate access to FP self-care interventions, particularly by targeting communities that lack access to hospital facility-based services such as market vendors [19].

The use of mobile phones has become a popular way of solving healthcare and related problems. Mobile phones can help in facilitating access to varieties of health information, interventions and diagnostics [2, 6]. Although there is some link between the use of mobile phones and willingness to use the technology for self-care interventions, limited research has been conducted in this area [2], particularly targeting underserved groups such as female market vendors [19, 22]. The use of mobile phones is seen as an innovative approach that could reveal and impact the utilization of modern FP interventions [23]. Previous studies have shown the application of mobile phones in numerous healthcare domains, including chronic diseases, skin diseases, noncommunicable diseases, and sexual and reproductive health services, among others [22, 24]. However, the use of mobile phones for health could depend on several conditions. These include ownership of phone, availability of airtime and mobile data, digital information literacy, issues of privacy and confidentiality, and awareness of the benefits of mobile phones and the information needs of users [3, 22]. In addition, the other critical condition that the use of digital health technologies for health depends on is the acceptance of digital health technologies by end-users [3].

A number of theories have been used to explain the acceptance of mHealth technologies. One of the theoretical frameworks found to be applicable for assessing human behaviour towards the potential acceptance or rejection of technology such as mobile phones for health is the technology acceptance model (TAM) [25, 27]. The TAM was developed by Fred Davis in 1989 and postulates that individual behaviours can be explained by two main factors, namely, perceived usefulness and perceived ease of use [26, 27]. Perceived ease of use is defined as ‘‘the degree to which an individual believes that using a particular technology would be free of physical and mental effort.’’, and perceived usefulness is ‘‘the degree to which an individual believes that using a particular system would enhance his or her performance.’’ [26, 28]. These two constructs were found to be useful predictors of mHealth [25]. In addition, we included other factors found in the literature that relate to the use of mHealth, such as digital skills and privacy [3, 14, 21–23]. We defined privacy as the degree to which the user believes that using mobile phones provides the privacy and confidentiality needed, and we defined skills as the degree to which a user believes she has the required skills to use mobile phones. In this study, we examined the attitudes of female market vendors of reproductive age towards the use of mobile phones and access to FP self-care interventions in Gulu city, northern Uganda. Specifically, we examined the relationships between ease of use of mobile phones, usefulness of mobile phones, privacy of using mobile phones, skills in using mobile phones, and access to FP self-care interventions.

## Methods

### Study design and setting

We used a cross-sectional survey design. The study was conducted in Gulu City Main Market from March 6th to March 9^th,^ 2023, and a total of 205 female market vendors aged 18–49 years responded. Gulu city’s main market is one of the largest markets in northern Uganda and is estimated to accommodate approximately 3000 market vendors at full capacity. The population of women of reproductive age in the market is estimated to be approximately 700 [29]. The government of Uganda has constructed similar markets across the country to boost trade in agricultural produce. The total number of vendors registered is approximately 20,000, of which more than 60% are women [21]. We targeted female market vendors because they lack access to hospital-based facilities due to the nature of their work, which requires their presence in the market [19].

### Population and sample

We recruited only female market vendors aged 18–49 years. We used Krejcie and Morgan’s (1970) table to determine a sample size of 248 from an eligible population of 700 vendors [30]. Simple random sampling was used for selecting participants. All 248 random numbers were generated using ^CalculatorSoup^® (https://www.calculatorsoup.com*)*, and participants were systematically recruited based on unique numbers that corresponded to their stall and lock-up shop numbers. Those who consented to participate were included in the study.

### Instrument and procedures

We used a structured researcher-administered questionnaire developed for this study (see additional file 1). The instrument comprised the participants’ background information, and four other constructs elicited information about attitudes towards the use of mobile phones and access to FP self-care interventions. The questions about perceived ease of use of mobile phones included 10 questions, 10 questions about perceived usefulness of mobile phones, 10 questions about perceived privacy with using mobile phones, 10 questions about perceived skills in using mobile phones, and 8 questions about access to FP self-care interventions. Each of the questions was measured on a 5-point attitudinal Likert-type ordinal scale ranging from 1 = strongly disagree, 2 = disagree, 3 = not sure, 4 = agree and 5 = strongly agree. The raw data collected through the instrument were entered and analysed using SPSS version 15. Before the final analysis, we checked the data for completeness and correctness using a frequency procedure. We also checked for internal consistency of the items in each of the constructs and generated Cronbach’s alpha values, as shown in Table 1. The alpha values for the five main variables were all greater than 0.7, indicating an acceptable range [31].

**Table 1:** Internal consistency of the questionnaire

Variable	No. of Item	Cronbach’s alpha value
Perceived ease of use of mobile phone	10	0.986
Perceived usefulness of mobile phone	10	0.974
Perceived privacy with using mobile phone	10	0.966
Perceived skills in using mobile phone	10	0.954
Access to FP self-care interventions	08	0.983

FP – Family planning

### Data analysis

Descriptive and inferential analyses were performed using SPSS version 15. Descriptive analysis using the frequency procedure was performed to display frequencies and percentages to describe participants’ background information (age, education level, marital status, types of phones used, and awareness of FP self-care interventions). Furthermore, descriptive analyses were performed using the frequency procedure to show the frequency, percentage, and mean to enable assessment of the use of mobile phones and access to FP self-care interventions. Attitudes were assessed on perceived ease of use of mobile phones, perceived usefulness of mobile phones, perceived privacy with using mobile phones, perceived skills in using mobile phones, and access to FP self-care interventions. Attitudes towards the study constructs were measured on a 5-point attitudinal Likert-type ordinal scale ranging from 1 = strongly disagree, 2 = disagree, 3 = not sure, 4 = agree and 5 = strongly agree. We used the mean to summarize and report participants’ attitudes for each of the constructs. For inferential analysis, we performed standard multiple regression statistics to examine the relationships between attitudes towards mobile phones (using perceived ease of use of mobile phones, perceived usefulness of mobile phones, perceived privacy with using mobile phones, perceived skills in using mobile phones, and access to FP self-care interventions). We used unadjusted regression and a significance level of 95% confidence intervals.

## Results

### Background information

Two hundred five (205) out of a sample of 248 participants responded to the study (response rate of 82.7%). In the Gulu Main Market, vendors are scheduled for market days outside the city (auction) from Monday – Friday. This affected contact with some target participants within the stipulated time for the study. However, the relatively good response rate was partly attributed to the use of a researcher-administered questionnaire.

**Table 2:** Background information of the respondents

Variable		Frequency	Percentage
Age category	18–21 years	16	7.8
	22–26 years	34	16.6
	27–31 years	53	25.9
	32–36 years	41	20
	37–41 years	33	16.1
	42–46 years	19	9.3
	47–51 years	9	4.4
Education level	Primary	75	36.6
	Secondary	89	43.4
	Certificate	16	7.8
	Diploma	7	3.4
	Degree	15	7.3
	Master Degree	0	0
	Others	3	1.5
Marital status	Married	96	46.8
	Single	59	28.8
	Separated	39	19
	Widow	11	5.4
Type of mobile phone used	Smartphone	112	54.6
	Feature Phones	93	45.4
Awareness of FP self-care interventions	Yes	147	71.7
	No	58	28.3

FP – Family planning

As presented in Table 2, one-quarter of the participants were aged 27–31 years (25.9%), followed by those aged 32–36 years (20%). Furthermore, nearly half of the participants indicated that they were at the secondary level of education (89, 43.4%), followed by those who had attained the primary level of education (75, 36.6%). Additionally, almost half of the participants were married women (96, 46.8%), followed by those who were single (59, 28.8%). In terms of the types of mobile phones used, more than half of the participants (112, 54.6%) used smartphones. Finally, our study also showed that the majority of participants (147, 71.7%) were aware of FP self-care interventions.

### Attitudes towards mobile phones and access to FP self-care interventions

As summarized in Table 3, attitudes were slightly positive about perceived ease of use (mean = 3.31) and perceived usefulness of mobile phones (mean = 3.30).

**Table 3:** Attitudes towards mobile phones and use for FP self-care interventions

Variable	Item	Mean	Attitude
Perceived ease of use of mobile phone	10	3.31	Slightly positive
Perceived usefulness of mobile phone	10	3.30	Slightly positive
Perceived privacy with using mobile phone	10	4.04	Strong positive
Perceived skills in using mobile phone	10	4.04	Strong positive
Use of mobile phone to access FP self-care interventions	08	3.18	Moderate

FP – Family planning

Furthermore, there were strong positive attitudes towards privacy (mean = 4.04) and skills associated with the use of mobile phones (mean = 4.04). However, participants had moderate attitudes towards access to FP self-care interventions (mean = 3.18) (see Appendix 1 Table 5, Appendix 2 Table 6, Appendix 3 Table 7, Appendix 4 Table 8, and Appendix 5 Table 9).

### Influence of attitudes towards use of mobile phones on access to FP self-care interventions

**Table 4:** Attitudes towards mobile phones and access to FP self-care interventions

Model		Unstandardized Coefficients		Standardized Coefficients	*t*	Sig.
		B	Std. Error	Beta	B	
1	(Constant)	-3.156	2.178		-1.449	0.149
	PMPS	0.208	0.061	0.222	3.419	0.001
	PEUoMP	0.278	0.066	0.360	4.200	0.000
	PUoMP	0.085	0.064	0.110	1.319	0.189
	PPUMP	0.201	0.065	0.192	3.102	0.002

*Note* R2 = 0.554 (55.4%)

Dependent Variable: Access to FP self-care interventions (AFPIs)

PMPS – Perceived mobile phone skills

PEUoMP – Perceived ease of use of mobile phone

PUoMP – Perceived usefulness of the mobile phone

PPUMP – Perceived privacy with using a mobile phone

As shown in Table 4, we performed a standard multiple regression between access to FP self-care intervention and attitudes towards the use of mobile phones. Our results showed a significant positive relationship between perceived ease of use of mobile phones and access to FP self-care interventions (Beta = 0.360, t = 4.200, *p* value = 0.000), between perceived mobile phone skills and access to FP self-care interventions (Beta = 0.222, t = 3.419, *p* value = 0.001), and between perceived privacy with using mobile phones and access to FP self-care interventions (Beta = 0.192, t = 3.102, *p* value = 0.002). On the other hand, we found a nonsignificant positive relationship between the perceived usefulness of using mobile phones and access to FP self-care interventions (Beta = 0.110, t = 1.39, *p* value = 0.189). Therefore, our results showed that participants with positive attitudes toward the ease of use of mobile phones, mobile phone skills, and privacy while using mobile phones, would likely access FP self-care interventions.

## Discussions

Our study examined the relationships between attitudes towards the use of mobile phones and access to FP self-care interventions among female market vendors of reproductive age in Gulu city, northern Uganda. Descriptively, we found that participants had positive to strong positive attitudes towards the use of mobile phones and moderate attitudes towards access to FP self-care interventions. In particular, positive attitudes were found for ease of use and usefulness of mobile phones, privacy provided while using mobile phones, and skills in using mobile phones. Our findings corroborate other studies where positive attitudes towards the use of mobile phones for accessing sexual and reproductive health services were reported [19]. A study by Feroz et al. [22]. , showed that women use mobile phones because the technology is useful for them and can support the information gap in FP interventions. Participants also felt comfortable with having greater privacy in using mHealth to access FP interventions [14]. Our study demonstrated that participants expressed knowledge and aptitude to learn how to use mobile phones. Others have indicated the ability to use more than one phone application, such as calls, video, the internet and messaging. They felt that the use of mobile phones protects their privacy and identity and is safe for personalized services. Furthermore, participants pointed out that using mobile phones is cost-effective [32]. Whereas participants showed positive attitudes towards the use of mobile phones, attitudes towards access to FP self-care interventions were moderate. It appears that, much as the majority of participants in our study (71.7%) indicated awareness of FP self-care interventions, they had not taken time to use mobile phones to access these interventions. Leahy et al. [12] argued that there is no guarantee that access to or favourable attitudes toward the use of mobile phones would automatically translate to the use of this technology for accessing self-care interventions. Therefore, it is important to promote the use and benefits of mobile phones for accessing FP interventions. Healthcare providers, health development partners, and the government are well positioned to lead these promotional activities. These efforts should be directed to communities with limited access to hospital-based facility services. Market vendors whose occupation requires their presence in the market [19] would benefit from such interventions.

Furthermore, we observed in our study that having positive attitudes towards the use of mobile phones would likely lead to access to FP self-care interventions. In particular, we found positive and significant associations between ease of use of mobile phones, skills in using mobile phones, privacy with using mobile phones, and access to FP self-care interventions. Our findings were supported by previous studies in which skills and privacy were found to be associated with access to FP self-care interventions and other healthcare-related services [14, 22, 33]. In addition, a study has shown that the perceived ease of use of mHealth is a sign of behavioural intentions towards technology for healthcare-related activities [26]. Whereas positive influences of ease of use, skills and privacy on access to FP self-care interventions were observed, an insignificant association was observed between the perceived usefulness of mobile phones and access to FP self-care interventions. This finding is consistent with that of Shemesh and Barnoy [26], who reported an insignificant influence of the perceived usefulness of mHealth on behavioural intentions to use the technology. In our study, all the participants indicated that they used mobile phones. Although we did not emphasize what participants used mobile phones for, the nonsignificant influence of perceived ease of use could be associated with the lack of use of mobile phones for accessing FP self-care interventions in this study. It is therefore not a guarantee that ownership or use of mobile phones for other purposes will translate to their use for healthcare purposes [12]. This therefore requires the alignment of interventions to improve mobile phone skills while promoting the benefits of the use of mobile phones for healthcare purposes. Healthcare practitioners, health development partners and the government can align their strategies to promote and popularize the use of mHealth, such as mobile phones, while focusing on improving access to FP interventions among women with limited access to hospital facility-based services in the Uganda.

### Strengths and limitations of this study

Our study used a cross-sectional design capable of only relating the study variables. Thus, no causality was assessed. Future studies can use the same or similar variables with other designs to determine causality. In addition, our study did not provide a sample size calculation for nonresponses, which might affect the generalizability of our findings to a wider catchment. Furthermore, the use of random sampling could have constrained the estimation of parameters for the different subgroups within the study population. Future studies should consider using stratified sampling to compare subgroups based on sex, age, education level and other factors. Additionally, the use of structured researcher-administered questionnaires with predetermined responses might have restricted participants from freely giving their opinions and might have produced biases since assistants were present beside participants. In conclusion, our tool generated only quantitative data. We recommend the use of a qualitative or mixed methods approach to study similar areas or other groups of women, such as teachers and forces (police and army), whose occupations could limit their ability to access hospital facility-based services.

## Conclusions

mHealth technologies such as mobile phones are revolutionizing the delivery of health and access to healthcare interventions. This study has demonstrated that positive attitudes towards the use of mobile phones could lead to access to FP self-care interventions among female market vendors of reproductive age in northern Uganda. Despite the positive attitudes towards the use of mobile phones, participants seemed to be uncertain about the usefulness of mobile phones for accessing FP self-care interventions. It is therefore important for healthcare practitioners, health development partners and the government to encourage and integrate the use of mHealth into regular FP self-care services and promotional activities while targeting underserved communities in Uganda.

### Electronic supplementary material

Below is the link to the electronic supplementary material.

Supplementary Material 1

Supplementary Material 2

Supplementary Material 3

Supplementary Material 4

Supplementary Material 5

## Data Availability

The data supporting the conclusions of this article are all included in the paper.

## Abbreviations

AFP: Access to FP self-care interventions
FP: Family planning
GUREC: Gulu University Research Ethics Committee
LMICs: Low- and middle-income countries
mHealth: Mobile Health
PEUoMP: Perceived ease of use of mobile phone
PMPS: Perceived mobile phone skills
PPUMP: Perceived privacy with using a mobile phone
PUoMP: Perceived usefulness of the mobile phone
TAM: Technology acceptance model
