# Supplementary material for: Attitudes of female market vendors of reproductive age towards use of mobile phones and access to family planning self-care interventions in Northern Uganda: a cross-sectional study
Source: BMC Med Inform Decis Mak. 2024 Jun 12;24:164. doi: 10.1186/s12911-024-02565-5 (PMC11167777; doi:10.1186/s12911-024-02565-5)
Supplement: Supplementary file 1 — Supplementary Material 1 [file 12911_2024_2565_MOESM1_ESM.docx]

**Supplementary Table 1: Use of mobile phone to access FP self-care interventions**

| Item | SD(1) | | D(2) | | NS(3) | | A(4) | | SA(5) | | Mean |
| --- | --- | --- | --- | --- | --- | --- | --- | --- | --- | --- | --- |
|  | *f* | *%* | *f* | *%* | *f* | *%* | *f* | *%* | *f* | *%* |  |
| I use mobile phone to access information on self-administration of injectables | 29 | 14 | 27 | 13 | 53 | 26 | 71 | 35 | 25 | 12 | 3.18 |
| I use mobile phone to access information on self-management of contraceptive | 25 | 12 | 28 | 14 | 54 | 26 | 73 | 36 | 25 | 12 | 3.22 |
| I use mobile phone to access information on self-screening with ovulation predictor | 24 | 12 | 29 | 14 | 68 | 32 | 60 | 29 | 24 | 12 | 3.15 |
| I use mobile phone to access information on home-based ovulation predictor kits | 25 | 12 | 31 | 15 | 66 | 32 | 56 | 27 | 27 | 13 | 3.14 |
| I use mobile phone to access information about female condoms | 22 | 11 | 39 | 19 | 52 | 25 | 60 | 29 | 32 | 16 | 3.2 |
| I use mobile phone to access information on self-testing for pregnancy | 23 | 11 | 39 | 19 | 50 | 24 | 50 | 25 | 41 | 20 | 3.24 |
| I use mobile phone to access information on over-the-counter contraceptive pills | 28 | 14 | 39 | 19 | 46 | 22 | 58 | 38 | 34 | 17 | 3.15 |
| I use mobile phone to seek health workers’ help on self-care interventions | 28 | 14 | 27 | 18 | 47 | 23 | 56 | 27 | 37 | 18 | 3.18 |
| **Total average score** |  |  |  |  |  |  |  |  |  |  | **3.18** |

Key: SD – Strongly disagree; D – Disagree; NS – Not sure; A – Agree; SA – Strongly agree
