# Supplementary material for: Attitudes of female market vendors of reproductive age towards use of mobile phones and access to family planning self-care interventions in Northern Uganda: a cross-sectional study
Source: BMC Med Inform Decis Mak. 2024 Jun 12;24:164. doi: 10.1186/s12911-024-02565-5 (PMC11167777; doi:10.1186/s12911-024-02565-5)
Supplement: Supplementary file 2 — Supplementary Material 2 [file 12911_2024_2565_MOESM2_ESM.docx]

**Supplementary Table 2: Ease of use of mobile phone**

| **Item** | **SD(1)** | | **D(2)** | | **NS(3)** | | **A(4)** | | **SA(5)** | | **Mean** |
| --- | --- | --- | --- | --- | --- | --- | --- | --- | --- | --- | --- |
|  | *f* | *%* | *f* | *%* | *f* | *%* | *f* | *%* | *f* | *%* |  |
| Knowledge in using mobile phone makes me access information | 17 | 8.3 | 44 | 21.5 | 39 | 19 | 66 | 32.2 | 39 | 19 | 3.32 |
| I understand the use of mobile phone to access information | 19 | 9.3 | 42 | 20.5 | 38 | 18.5 | 62 | 30.2 | 44 | 21.5 | 3.34 |
| It is easy for me to use mobile phone to access information | 20 | 9.8 | 43 | 21 | 38 | 18.5 | 63 | 30.7 | 41 | 20 | 3.3 |
| I need less effort to use mobile phone to access information | 20 | 9.8 | 48 | 23.4 | 39 | 19 | 61 | 29.8 | 40 | 19.5 | 3.27 |
| I took less time to learn how to use mobile phone to access information | 19 | 9.3 | 48 | 23.4 | 37 | 18 | 57 | 27.8 | 44 | 21.5 | 3.29 |
| It is easy to operate mobile phone to access health information | 18 | 8.8 | 45 | 22 | 42 | 20.5 | 55 | 26.8 | 45 | 26.8 | 3.31 |
| It is flexible for me to use mobile phone to access information | 17 | 8.3 | 45 | 22 | 41 | 20 | 56 | 27.3 | 46 | 22.4 | 3.34 |
| I took less training to use mobile phone to access information | 17 | 8.3 | 45 | 22 | 44 | 21.5 | 56 | 27.3 | 43 | 21 | 3.31 |
| I remembered how to use mobile phone to access health information | 23 | 11.2 | 39 | 19 | 45 | 22 | 49 | 23.9 | 49 | 23.9 | 3.3 |
| It is easy to use mobile phone to consult health workers | 23 | 11.2 | 42 | 20.5 | 39 | 19 | 48 | 23.4 | 53 | 25.9 | 3.32 |
| **Total average score** | | | | | | | | |  |  | **3.31** |

Key: SD – Strongly disagree; D – Disagree; NS – Not sure; A – Agree; SA – Strongly agree
