# Supplementary material for: Attitudes of female market vendors of reproductive age towards use of mobile phones and access to family planning self-care interventions in Northern Uganda: a cross-sectional study
Source: BMC Med Inform Decis Mak. 2024 Jun 12;24:164. doi: 10.1186/s12911-024-02565-5 (PMC11167777; doi:10.1186/s12911-024-02565-5)
Supplement: Supplementary file 3 — Supplementary Material 3 [file 12911_2024_2565_MOESM3_ESM.docx]

**Supplementary Table 3: Usefulness of mobile phone**

| **Item** | **SD(1)** | | **D(2)** | | **NS(3)** | | **A(4)** | | **SA(5)** | | **Mean** |
| --- | --- | --- | --- | --- | --- | --- | --- | --- | --- | --- | --- |
|  | *f* | *%* | *f* | *%* | *f* | *%* | *f* | *%* | *f* | *%* |  |
| It is useful to use mobile phone to access health information | 14 | 6.8 | 42 | 20.5 | 37 | 18 | 51 | 24.9 | 61 | 29.8 | 3.5 |
| Use of mobile phone increases my chances to get information | 14 | 6.8 | 54 | 26.3 | 38 | 18.5 | 61 | 29.8 | 38 | 18.5 | 3.27 |
| Use of mobile phone makes me access quality information | 15 | 7.3 | 53 | 25.9 | 38 | 18.5 | 63 | 30.7 | 36 | 17.6 | 3.25 |
| Use of mobile phone makes me access accurate information | 16 | 7.8 | 54 | 26.3 | 35 | 17.1 | 60 | 29.3 | 40 | 19.5 | 3.26 |
| Use of mobile phone makes me access reliable information | 14 | 6.8 | 56 | 27.3 | 35 | 17.1 | 61 | 29.8 | 39 | 19.0 | 3.27 |
| Use of mobile phone makes me make good decisions. | 15 | 7.3 | 54 | 26.3 | 36 | 17.6 | 51 | 24.9 | 49 | 23.9 | 3.32 |
| Use of mobile phone enable me access timely information | 16 | 7.8 | 53 | 25.9 | 36 | 17.6 | 51 | 24.9 | 49 | 23.9 | 3.31 |
| Use of mobile phone makes me have control over information | 16 | 7.8 | 48 | 23.4 | 39 | 19 | 61 | 29.8 | 40 | 19.5 | 3.39 |
| I use mobile phone to communicate with health workers | 25 | 12.2 | 44 | 21.5 | 37 | 18 | 53 | 25.9 | 46 | 22.4 | 3.25 |
| I use mobile phone to access promotional health information | 28 | 13.7 | 42 | 20.5 | 36 | 17.6 | 61 | 29.8 | 36 | 22.4 | 3.19 |
| **Total average score** |  |  |  |  |  |  |  |  |  |  | **3.30** |

Key: SD – Strongly disagree; D – Disagree; NS – Not sure; A – Agree; SA – Strongly agree
