# Supplementary material for: Attitudes of female market vendors of reproductive age towards use of mobile phones and access to family planning self-care interventions in Northern Uganda: a cross-sectional study
Source: BMC Med Inform Decis Mak. 2024 Jun 12;24:164. doi: 10.1186/s12911-024-02565-5 (PMC11167777; doi:10.1186/s12911-024-02565-5)
Supplement: Supplementary file 4 — Supplementary Material 4 [file 12911_2024_2565_MOESM4_ESM.docx]

**Supplementary Table 4: Privacy provided while using mobile phone**

| **Item** | **SD (1)** | | **D(2)** | | **NS (3)** | | **A(4)** | | **SA(5)** | | **Mean** |
| --- | --- | --- | --- | --- | --- | --- | --- | --- | --- | --- | --- |
|  | *f* | *%* | *f* | *%* | *f* | *%* | *f* | *%* | *f* | *%* |  |
| Use of mobile phone protects my privacy | 8 | 3.9 | 13 | 6.3 | 12 | 5.9 | 89 | 43.4 | 83 | 40.5 | 4.1 |
| Use of mobile phone protects my identity | 10 | 4.9 | 11 | 5.4 | 13 | 6.3 | 88 | 42.9 | 83 | 40.5 | 4.09 |
| Use of mobile phone protects my security | 8 | 3.9 | 16 | 7.8 | 11 | 5.4 | 92 | 44.9 | 78 | 38 | 4.05 |
| Use of mobile phone makes me remain confidential | 6 | 2.9 | 17 | 8.3 | 13 | 6.3 | 90 | 43.9 | 79 | 38.5 | 4.07 |
| Use of mobile phone makes me avoid stigmatization | 8 | 3.9 | 16 | 7.8 | 26 | 12.7 | 116 | 56.6 | 39 | 19 | 3.79 |
| I am not fearful when I use mobile phone | 4 | 2.0 | 17 | 8.3 | 15 | 7.3 | 88 | 42.9 | 81 | 39.5 | 4.1 |
| I remain anonymous when I use mobile phone | 7 | 3.4 | 15 | 7.3 | 24 | 11.7 | 102 | 49.8 | 57 | 27.8 | 3.91 |
| My autonomy is protected when I use mobile phone | 8 | 3.9 | 14 | 6.8 | 20 | 9.8 | 90 | 43.9 | 73 | 35.6 | 4.0 |
| Use of mobile phone allows me access personalized services | 7 | 3.4 | 11 | 5.4 | 19 | 9.3 | 79 | 38.5 | 89 | 43.4 | 4.13 |
| I feel safe when I use mobile phone for health communication | 8 | 3.9 | 10 | 4.9 | 18 | 8.8 | 74 | 36.1 | 95 | 46.3 | 4.16 |
| **Total average score** |  |  |  |  |  |  |  |  |  |  | **4.04** |

Key: SD – Strongly disagree; D – Disagree; NS – Not sure; A – Agree; SA – Strongly agree
