# Supplementary material for: Attitudes of female market vendors of reproductive age towards use of mobile phones and access to family planning self-care interventions in Northern Uganda: a cross-sectional study
Source: BMC Med Inform Decis Mak. 2024 Jun 12;24:164. doi: 10.1186/s12911-024-02565-5 (PMC11167777; doi:10.1186/s12911-024-02565-5)
Supplement: Supplementary file 5 — Supplementary Material 5 [file 12911_2024_2565_MOESM5_ESM.docx]

**Supplementary Table 5: Skills in using mobile phone**

| Item | SD (1) | | D(2) | | NS (3) | | A(4) | | SA (5) | | Mean |
| --- | --- | --- | --- | --- | --- | --- | --- | --- | --- | --- | --- |
|  | *f* | *%* | *f* | *%* | *f* | *%* | *f* | *%* | *f* | *%* |  |
| I know how to make calls using mobile phone | 4 | 2.0 | 10 | 4.9 | 9 | 4.4 | 58 | 28.3 | 124 | 60.5 | 4.4 |
| I know how to send messages using mobile phone | 5 | 2.4 | 14 | 6.8 | 11 | 5.4 | 57 | 27.8 | 118 | 57.6 | 4.31 |
| I know how to open messages using mobile phone | 6 | 2.9 | 13 | 6.3 | 11 | 5.4 | 55 | 36.8 | 120 | 58.5 | 4.32 |
| I know how to install applications on mobile phone | 23 | 11.2 | 16 | 7.8 | 15 | 7.3 | 58 | 28.3 | 93 | 45.4 | 3.89 |
| I know how to use videos on mobile phone | 15 | 7.3 | 24 | 11.7 | 14 | 6.8 | 65 | 31.7 | 87 | 42.4 | 3.9 |
| I know how to use social media using mobile phone | 20 | 9.8 | 23 | 11.2 | 13 | 6.3 | 57 | 27.8 | 92 | 44.9 | 3.87 |
| I know how to use Internet using mobile phone | 19 | 9.3 | 24 | 11.7 | 14 | 6.8 | 60 | 29.3 | 88 | 42.9 | 3.85 |
| I know how to keep contacts on mobile phone | 4 | 2.0 | 14 | 6.8 | 14 | 6.8 | 56 | 27.3 | 117 | 57.1 | 4.31 |
| I know how to look for useful health information on my phone | 11 | 5.4 | 21 | 10.2 | 20 | 9.8 | 57 | 27.8 | 96 | 46.8 | 4.0 |
| I know how to use and send messages using my phone | 13 | 6.3 | 20 | 9.8 | 20 | 9.8 | 54 | 26.3 | 98 | 47.8 | 4.0 |
| **Total average score** |  |  |  |  |  |  |  |  |  |  | **4.04** |

Key: SD – Strongly disagree; D – Disagree; NS – Not sure; A – Agree; SA – Strongly agree
